# Progress and controversies in developing cancer vaccines

**DOI:** 10.1186/1479-5876-3-18

**Published:** 2005-04-29

**Authors:** Craig L Slingluff, Daniel E Speiser

**Affiliations:** 1 University of Virginia, Charlottesville, Virginia, USA; 2 Ludwig Institute for Cancer Research, Lausanne Branch, Lausanne, Switzerland

## Abstract

Immunotherapy has become a standard approach for cancer management, through the use of cytokines (eg: interleukin-2) and monoclonal antibodies. Cancer vaccines hold promise as another form of immunotherapy, and there has been substantial progress in identifying shared antigens recognized by T cells, in developing vaccine approaches that induce antigen-specific T cell responses in cancer patients, and in developing new technology for monitoring immune responses in various human tissue compartments. Dramatic clinical regressions of human solid tumors have occurred with some cancer vaccines, but the rate of those responses remains low. This article is part of a 2-part point:counterpoint series on peptide vaccines and adoptive therapy approaches for cancer. The current status of cancer vaccination, and associated challenges, are discussed. Emphasis is placed on the need to increase our knowledge of cancer immunobiology, as well as to improve monitoring of cellular immune function after vaccination. Progress in both areas will facilitate development of effective cancer vaccines, as well as of adoptive therapy. Effective cancer vaccines promise to be useful for treatment and prevention of cancer at low cost and with low morbidity.

## Cancer immunotherapy: transition from nonspecific to specific immunotherapy

There is broad appeal for the concept of treating cancer with the immune system. Early anecdotal experiences over 100 years ago suggested that induction of generalized immune activation, by a bacterial infection, could induce regression of solid human cancers in a small subset of patients [1,2]. However, efforts to generalize this finding by treating patients with bacterial agents (e.g.: Bacille Calmette-Guerin, BCG) were disappointing [3]. Subsequent efforts were to vaccinate with cancer cell preparations to induce immune responses more specifically against cancer antigens that had not yet been defined. These included whole cell vaccines, cancer cell lysates, and cultured cell supernatants [4-8]. The molecular identity of cancer-specific antigens was sought over several decades, with most of the work focusing on melanoma. Initially, numerous cell surface antigens were identified by serologic methods in mice [9]. Vaccination against those antigens can induce specific antibodies [10]. However, a recent clinical trial of vaccination against one such antigen (the ganglioside GM2) had a negative result in terms of clinical outcome [11]. The potential of vaccines for induction of anti-tumor antibodies has not been fully explored, and deserves further investigation. However, in recent years, substantial effort has been directed at defining antigenic targets for CD8+ cytotoxic T lymphocytes (CTL), leading to new vaccine strategies designed to induce antigen-specific CTL using these antigens.

## Preclinical models of tumor vaccines: role of CD8+ and CD4+ T cells in tumor protection

In murine studies, cell-based tumor vaccines can protect against cancer progression and can lead to regression of early established tumors. The protective immunity induced by syngeneic tumor vaccines appears to be mediated most directly by T-cells, and in many studies, depletion of CD8^+ ^T cells abrogates the protective effect of syngeneic tumor cell vaccines [12], suggesting cytotoxic T-cells are critical to that protective immunity. In some studies, however, depletion of CD4^+ ^T-cells also abrogates all or part of the protective immune response to vaccines [13]. Furthermore, adoptive therapy with CD4^+ ^T-cells can induce tumor protection in some model systems [14]. Thus, the protective immunity induced by syngeneic tumor cell vaccines appears to be mediated both by CD8^+ ^T-cells and by CD4^+ ^T-cells. These findings directed efforts toward identifying the molecular nature of tumor antigens recognized by CD8 and CD4 T cells. It was only in the last 1–2 decades that the nature of these antigens became known [15]. It was discovered that short peptides from cellular proteins were presented in association with cell-surface MHC molecules, and that these peptides represented epitopes for these T cells.

## Molecular definition of tumor antigens recognized by T-cells

In the late 1980s, it was found that melanomas expressed shared antigens recognized by CD8^+ ^cytotoxic T lymphocytes (CTL) [16]. Subsequent studies beginning in the 1990s defined the molecular nature of some of these antigens [17-22]. The peptides recognized by cytotoxic (CD8^+^) T-cells are typically 8–10 amino acids long and are presented in association with Class I MHC molecules. The peptides recognized by helper (CD4^+^) T-cells are usually longer (generally 13–18 amino acids in length, although peptide elution studies have indicated no apparent restriction on peptide length) and are presented in association with Class II MHC molecules. For melanoma, the melanocytic differentiation proteins (MDPs) and the cancer-testis antigens (CTAs) are the most common source proteins for these defined shared peptide antigens. Now, a large number of peptide epitopes recognized by melanoma-reactive human CTL and helper T-cells are known (reviewed in [23,24], making it possible to design vaccines using these antigens. At least as importantly, evaluation of T-cell responses to these defined antigens is now possible, and may permit evaluation of the immune responses induced by vaccine strategies, and to dissect the immune response. As outlined below, it has become clear that this approach can aid in optimizing vaccines. Peptide vaccines provide the unique opportunity to evaluate the T cell responses specifically to defined immunogens.

## Application of defined antigens to tumor vaccines

Peptide epitopes for melanoma-reactive cytotoxic T-cells were first identified in 1991, and epitopes for melanoma-reactive helper T cells have been identified in recent years. Some of these agents have been employed in experimental melanoma vaccines over the past 10 years or less. Peptide vaccines have theoretical and practical appeal, but also have certain drawbacks, as summarized in Tables 1 and 2.

**Table 1:** Practical and Theoretical Advantages of Peptide vaccines for cancer

**Characteristic**	**Detail**	**Advantage vs**	
		
		**Tumor cell antigen sources**	**adoptive cellular therapy**
Pure	Avoid tolerizing cellular antigens; exclude normal protein, avoid autoimmunity.	**X**	
Processed	Avoid effects of immunoproteasome	**X**	
Cheap	Feasible to study without corporate support	**X**	**X**
Easier	Lower regulatory hurdles	**X**	**X**
Evaluable	Excellent cancer vaccine model, allowing direct evaluation of response to the specific immunogen	**X**	
Modifiable	Create synthetic peptides better than native peptides	**X**	
Immunogenic	Induce T cell responses in patients		
Combinable	Multipeptide vaccines may mimic immune effects of whole cell vaccines.		

**Table 2:** Limitations of peptide vaccines

• Limited by MHC restriction.
• Unique individual tumor-specific antigens difficult to include.
• Rapid degradation in vivo.
• Heterogeneity of tumor antigen expression.
• Ignorance. We don't yet know how best to vaccinate with them. *
• Clinical responses have been rare in most series (with peptide or any vaccine alone).*

* The last two points apply equally to practically all T cell vaccines, not just peptide vaccines.

With peptide vaccines, it has been possible to generate antigen-specific T cells at frequencies of 0.1% to greater than 2% percent of circulating CD8 T cells in many individuals [[25-29], and unpublished results]. However, when vaccines contain only single peptides, or small numbers of peptides, targeting CD8 T cells responses only, low clinical response rates have been observed [30]. In reality, that should not be surprising, especially in the setting of advanced tumor burden. Antigenic heterogeneity is the rule in tumor deposits. Adoptive therapy with T cell clones specific for a single antigen has led to eradication of melanoma cells expressing that antigen, but the tumors have not regressed, because of the persistence of antigen-loss variants [31]. Furthermore, T cells infiltrating tumor deposits are commonly found to be anergic or poorly responsive to antigenic stimulation, leading to the perception that the tumor microenvironment is hostile to the T cell response [32]. Effective immune therapy will require induction of T cell responses to multiple antigens simultaneously, and promotion of T cell activity in tumor tissue. Additional approaches to block immunoregulatory mechanisms may well also be needed for immune therapy to be successful.

### Is adoptive immunotherapy more fashionable than cancer vaccines?

Recent clinical successes in one study with adoptive T cell therapy in patients with metastatic melanoma have heightened enthusiasm for adoptive therapy [33]. In the wake of this renewed enthusiasm for adoptive T cell therapy, it has been stated that current peptide vaccines have failed [30]. Furthermore, a corollary argument is surfacing, that peptide vaccines (or other active specific immunotherapy for cancer) may not be worthy of continued investigation. This could not be more wrong. Perhaps the greatest failure of the tumor immunology research community is its reliance on fashion. Historically, encouraging early results with various immune therapies have induced great enthusiasm, followed soon thereafter by dashed hopes as the therapy proves not to be as effective as originally hoped. A lesson can be learned from the failures and successes of immunotherapy with monoclonal antibodies. In the 1970s and early 1980s, monoclonal antibodies were popularly considered magic bullets, and antibody therapy was in high fashion. Subsequently, in the 1980s, numerous therapeutic clinical trials with monoclonal antibodies led to very disappointing results. Consequently, monoclonal antibody therapy fell out of fashion. However, some persistent investigators focused on studying antibodies with certain specificities and on learning how to overcome HAMA reactions by humanizing monoclonal antibodies. The result has become common knowledge: multiple monoclonal antibodies are now used for several FDA approved therapies against cancer, such as herceptin (anti-Her-2/neu) and Rituximab (anti-CD20), and more recently Avastin (anti-VEGF). These successes took decades, but they now have firmly established immune therapy as a standard treatment option for multiple cancers. The lesson from this history is that one should persist in developing therapeutic approaches as long as they are promising and are built on continuous progress in the understanding of pathophysiological mechanisms. T cell immunotherapy of solid tumors is still in its experimental phase. Investigators in this field can and will bring together innovative tools and scientific reasoning in order to maximize the likelihood that the next generation of cancer vaccines will have therapeutic value.

Similarly, adoptive therapy approaches have been studied for many decades, with many false starts and failures prior to the current exciting results. The recent successes with adoptive therapy are welcome and offer promise for further development. However, as with cancer vaccines, there remains much work to optimize adoptive therapy.

The particular adoptive therapy study cited above is a modification of prior adoptive therapy approaches. Early enthusiasm for adoptive therapy with lymphokine-activated killer (LAK) cells in the 1980s was based on similar successes at the NCI, but subsequent multicenter investigations suggested that all or most of the therapeutic effect associated with LAK cell therapy could be mimicked by systemic therapy with high-dose IL-2 alone [34-37]. Subsequent studies with adoptive transfer of tumor-infiltrating lymphocytes (TIL) expanded *ex vivo *in IL-2 were associated with clinical regressions in 55% of patients in early studies [38], but this has largely been abandoned due to failure to maintain response rates that were convincingly better than that expected from high dose IL-2 alone [39,40]. The new approach to adoptive therapy at the NCI involves peripheral lymphoablation followed by adoptive transfer of TIL expanded *ex vivo *after selection for tumor lytic potential [33,41,42]. It is currently unclear whether the improved results with this combination therapy are due primarily to the lymphoablation, the adoptive transfer, or the type of T cells expanded for the adoptive transfer. Also, the high rate of objective clinical regressions in the current NCI experience (51%) is very similar to the high rate reported in prior NCI studies, which were not maintained in subsequent experience (Table 3).

**Table 3:** Rates of clinical tumor regression in studies of adoptive transfer of tumor-reactive lymphocytes

**Type of therapy**	**Initial rate of objective responses**	**Subsequent rate of objective responses**	**Conclusion**
LAK cell therapy + high-dose (HD) IL2	44% (11/25) [ref 34]	22% (23/106) [ref 35]	Response rate not better than HD IL2 alone (28 vs 22%), but trend toward improved survival with LAK+IL2 for melanoma (p = 0.064) [refs 36,37]
TIL therapy + HD IL2	55% (11/20) [ref 38]	22% (9/41) [ref 39]	Not better than HD IL2 alone [ref 39]. Median duration of partial responses 4 months [ref 40].
Selected TIL therapy after lymphoablation + HD IL2	51% (18/35) [ref 33]	Pending	Results preliminary

### Is it all about quantity or about quality?

One of the major arguments for use of adoptive cellular immune therapy for cancer is that it can achieve much higher numbers of circulating CD8 cells with anti-tumor specificity. Certainly it is true that patients treated with lymphoablation and adoptive TIL therapy plus high dose IL-2 have had extremely high numbers (and frequencies) of tumor-antigen specific T cells in circulation, with over 90% of circulating CD8 cells reacting to the immunodominant HLA-A2 restricted MART-1/MelanA antigen in one patient, and with a large proportion of patients having more than 10% of circulating CD8 cells with anti-tumor specificity [42].

A major observation is that the generation of high numbers of circulating anti-tumor CD8 T cells is insufficient to induce clinical tumor regressions in about half of patients, and is often insufficient to control melanoma completely in the large majority of patients. It can safely be concluded, thus, that factors other than the number of anti-tumor CD8 T cells affect immune control of cancer. These factors are being elucidated gradually, and they are the primary obstacles against which the next 5–10 years of translational and clinical research in immune therapy need to be targeted.

These obstacles to success of adoptive transfer therapies are the same that interfere with the clinical efficacy of cancer vaccines. Some of the obstacles to immunologic control of tumor progression are listed in Table 4. It is far more important for investigators in immunotherapy and cancer immunology to join forces in identifying and overcoming these factors than for us to argue whether peptide vaccines, viral vaccines, adoptive transfer, or other immunotherapy approaches are superior or inferior to others.

**Table 4:** Known or possible obstacles to immunologic control of tumor progression, which impact on both active immunotherapy (cancer vaccines) and adoptive immunotherapy.

1) Expression of tumor antigens in the absence of costimulatory molecules on tumor cells, leading to tolerance
2) Chronic antigen exposure, leading to upregulation of immuno-regulatory mechanisms
a) CTLA4 expression
b) Accumulation of regulatory T cells in the tumor microenvironment
3) Downregulation of MHC molecule expression by tumor cells
4) Downregulation of tumor antigen expression by tumor cells
5) Secretion of anti-inflammatory cytokines by tumor cells or tumor-associated stroma
a) IL-10
b) TGF-β
c) Others
6) Expression of enzymes in the tumor microenvironment that interfere with T cell function
a) Arginase
b) Indoleamine 2,3-dioxygenase (IDO)
7) Propogation of a tumor microenvironment that is hostile to T cell activation
a) Immunoregulatory function of dendritic cells
b) Anergic tumor-infiltrating lymphocytes
8) Tumor-associated VEGF and other neovascularity-enhancing mechanisms may have immunoregulatory properties as well.
9) Homeostatic mechanisms in the host may limit expansion of tumor-specific T cell responses, and may limit expansion and persistence of tumor-specific T cell responses.
10) Resistance of tumor cells to apoptosis
11) Elaboration of compounds associated with tumor necrosis, that inhibit anti-tumor immunity locally

Some patients enrolled in peptide vaccine studies have had marked expansion of antigen-reactive CD8+ T cells, with 5–10% of circulating CD8 cells reactive to antigen in some cases, and over 1% reactive to antigen in many cases [25-29]. While it is worthwhile to induce further expansion of T cells after cancer vaccines, it is likely that the quality of the immune response, rather than simply its magnitude, is critical to the success of immune therapy. Several approaches for improving immunotherapy with cancer vaccines need to be pursued, as listed in Table 5.

**Table 5:** Potential avenues for improving therapeutic value of cancer vaccines

**Obstacle**	**Potential solution**	**Status**
Heterogeneity of antigen expression	Multi-antigen vaccines	12 peptide vaccine induces T cell responses in 100% of patients. Peptide competition for MHC binding does not inhibit immunogenicity [ref 43]
MHC downregulation on tumor cells	Targeting peptides associated with multiple MHC molecules	Being investigated in many centers
Failure of T cells induced in the periphery with vaccines to expand in the tumor microenvironment (inadequate memory)	Addition of melanoma (or other cancer) associated helper peptides in vaccines [refs 24, 44]	Early data inadequate to address the question refs [45–47]. Data in the HIV setting supports this approach [ref 48.] ECOG 1602 trial will address the questions with a cocktail of 6 melanoma helper peptides.
Increased regulatory T cells in patients with advanced cancer, and in tumor microenvironment	Inhibition of T reg function (anti-CTLA4 antibody); specific depletion of CD25+ regulatory T cells (Ontak); depletion of regulatory cells with chemotherapy (eg: cytoxan)	Clinical trials with all of these agents are underway.
Limited expansion of antigen-specific T cells after vaccination	Pre-vaccine lymphodepletion to allow vaccination in the setting of naturally induced cytokines supporting homeostatic proliferation (eg IL7 and IL15)	Studies are being designed to address this approach
T cells induced by vaccination may not be activated effector cells	Increase adjuvant function, perhaps by use of Toll-like receptor agonists	CpGs and other TLR agonists being investigated as adjuvants [29]. Randomized phase II trials with immunologic endpoints needed.

### Proof of principle of vaccines for cancer

The current manuscript is focused primarily on vaccine therapy, especially peptide vaccines, for solid tumors such as melanoma. However, a very important paradigm of cancer immunotherapy should be mentioned in this discussion. For those cancers whose primary etiologic factor is a known viral infection, vaccination against infection with that virus promises to have significant oncologic value. Specific examples are listed in Table 6.

**Table 6:** Virally-induced cancers subject to control by vaccines.

**Cancer histology**	**Etiologic virus**	**Vaccine strategy**	**Current use**	**Clinical value**
Hepatoma	Hepatitis B	Protein subunit vaccine	In common use for high-risk populations.	Protection against Hepatitis B infection is prolonged after three vaccines. Worldwide protection against hepatoma may have dramatic impact.
Cervical adenocarcinoma	Human Papilloma Virus	Viral and other vaccines against E6 and E7	Strong evidence for efficacy in certain populations	Likely will protect against cancer, especially for patients without access to Pap smears
Burkitt's lymphoma, Nasopharyngeal cancer	Epstein-Barr Virus	Some T cell antigens identified	Vaccines would have to be administered very early in life	Untested.

There are differences between these clinical settings, where vaccines may prevent cancer by preventing the causative viral infection, and the more common scenario where vaccines are being considered to treat patients already diagnosed with cancer. The latter clinical setting represents chronic (vs. acute) antigen exposure and the reality that a cancer that progresses clinically has likely developed one or more mechanisms of immune escape or tolerance. Also, cancer progression commonly is associated with antigenic heterogeneity, which complicates the development of successful multi-antigen immunotherapy. However, the clinical and immunologic successes of anti-idiotype vaccines for some B cell lymphomas show that vaccines can induce protective immunity against a defined tumor-specific antigen, even in the setting of prior chronic antigen exposure [49,50]. Where the antigen is integral to the malignant cell, targeting that antigen can have encouraging clinical results.

Ultimately, the ideal cancer vaccine will be effective at inducing protective immunity, and will be safe enough to administer early in life before the initial carcinogenic events. Cancer vaccines, but not adoptive cellular therapy, hold out the prospect of being useful for cancer prevention on a wide scale.

## Objective clinical responses in patients enrolled in experimental melanoma vaccine trials

In numerous published clinical trial results with cancer vaccines, one or more objective clinical tumor regressions have been observed. Though the overall objective response rate is low [30], even these infrequent clinical responses are proof of principle of cancer vaccines. Most current vaccines target only one or a few cancer antigens, restricted usually by just one MHC molecule. Since antigenic heterogeneity is the hallmark of cancer, it is most remarkable that these simple vaccines can lead to clinical regressions in any patients. The majority of current vaccines also target only CD8+ T cells and largely ignore CD4+ T cell responses, and responses of the innate immune system. Again, considering how simplistic the early peptide-based vaccines are, it is remarkable, and even encouraging, that they have been associated with any clinical tumor regressions. Some published studies have reported the proportion of patients with regressions of even just one lesion, and thus describe a higher proportion of clinical tumor regression than would be reported using RECIST criteria. However, a summary review of the NCI experience with vaccines and of the global experience with antigen-specific cancer vaccines, reveals that objective clinical response rates globally are in the range of 3–4% with recent cancer vaccines [30]. While this is certainly low, it is relevant that reported response rates with approved systemic therapy are only 12% for DTIC (dacarbazine), 11% for CVD (cisplatin, vincristine, dacarbazine), 16% for high-dose interleukin 2, and 17% for biochemotherapy [51-53]. Considering the low toxicity of peptide vaccines, an argument can be made that even current cancer vaccines have a prospect of clinical benefit for patients that rivals that of approved therapies, when one considers the risk:benefit ratio.

### Monitoring

One of the arguable values of adoptive therapy is the ability to enrich or to deplete the cellular reagents and to define the specificity of the T cells used for therapy. With vaccines, it is not possible to select particular lymphocyte populations from the patient directly. However, the compartments of the immune system are natural environments for optimal expansion of T cells and for the complex interplay among innate and adaptive immune mechanisms. It is presumptuous to believe that our understanding of this complexity and our technologies are adequate to allow us to recreate optimal immune effectors *in vitro *and to expect them to perform as we desire upon re-infusion. However, it is possible in patients on clinical trials, to enrich for specific effectors by vaccination with defined antigens, and to measure their responses to each antigen simultaneously, in various compartments (eg: lymph node, blood, and tumor) [27,32,43,54]. Furthermore, manipulations can be performed *in vivo*, to enrich or to deplete certain T cell subsets. Reagents exist for depletion of regulatory T cells (Ontak), for depletion of T cells (OKT3) or B cells (Rituximab), and there is increasing evidence that numerous cytotoxic chemotherapy agents have immunomodulatory effects that may be useful for augmentation of immunotherapy. Our challenge is to characterize these agents and their effects on development of protective immunity in patients treated with cancer vaccines.

All such studies require careful immune monitoring, both to assess the effects of immune modulations over time, and to determine whether such changes are useful and evaluable. We would like to point out that surrogate endpoints for vaccine efficacy should be re-emphasized, despite some current sentiment to the contrary. For the development of new generation vaccines, we must rely on knowledge derived from basic research. In infectious diseases, it is well established that antigen specific lymphocytes must be activated substantially for successful (i.e. protective) vaccination. Consequently, assessing responses of antigen specific lymphocytes is an important step in the evaluation of novel vaccines.

There are a number of new techniques permitting investigators to dissect T cell responses *ex vivo*. It is now possible to determine molecular features of human T cell responses in great detail, going much beyond what is usually done to assess T cells in animal models [55-57]. Economical and ethical considerations require that one takes maximal advantage by studying each patient in depth. Moreover, many issues in modern vaccinology must be assessed specifically in humans, since species differences do not allow to draw direct conclusions from experimental models.

It is generally accepted that a protective T cell response includes T cells with high avidity T cell receptors, with expression of effector molecules and function, and with appropriate homing capability. Such features can and need to be determined by analyzing patients' T cells *ex vivo *before and after vaccine therapy, allowing evaluation of the potential value of a given vaccine. Many new vaccine candidates are being proposed to treat cancer patients. The scientific community is well advised to use biological readouts extensively in order to assess thoroughly the T cells from study patients. By doing so, one can rapidly eliminate useless approaches and promote good vaccine components for further development.

### Summary

Immune therapy of cancer may take many forms, specific or non-specific, adoptive or active, and may target antibody, T cell, and innate immune mechanisms. Each of these approaches has proven or potential value, and the complexity of the host: tumor relationship is such that a narrow focus on a single immunotherapy strategy is likely to fail. Adoptive T cell immunotherapy studies have provided strong proof of principle that antigen-specific CD8+ T cell responses to cancer can mediate dramatic cancer regressions. However, adoptive therapy is cumbersome and expensive, and difficult in the current regulatory environment. Vaccines, on the other hand, are more readily adaptable for therapy outside of highly specialized centers. In particular, peptide vaccines are easily produced, standardized, and administered. The current appeal of adoptive therapy is that antigen-specific T cells can be expanded and activated at high numbers *ex vivo*, more readily than they can be expanded *in vivo *in cancer patients. However, we argue that the lesion in current approaches to cancer vaccine therapy is our poor understanding of the mechanisms that limit expansion, activation, and effector function of tumor-antigen specific T cells. Bypassing this process by use of adoptive therapy is a reasonable short-term effort, but ultimately to advance the field of tumor immunology and immunotherapy it will be critical to elucidate the immunobiology of the host-tumor relationship. Appropriate design of cancer vaccines using multiple antigens should be combined with careful monitoring of T cell expansion and T cell function. Optimally, immune monitoring should be performed in multiple compartments (peripheral blood, tumor tissue, lymph nodes). The next wave of investigation in cancer immunotherapy has begun, and will include combination therapies designed to activate innate and adaptive immunity simultaneously and to down-modulate tumor-associated immune regulation. Vaccines with defined antigens are ideal for investigations of this type.
